# Childhood Neglect and Bipolar Disorder: A Systematic Review, Meta-Analysis And Meta-Regression

**DOI:** 10.1192/j.eurpsy.2026.10227

**Published:** 2026-07-31

**Authors:** M. Merola, S. Carletto, L. Ostacoli, F. Oliva

**Affiliations:** 1 Department of Clinical and Biological Sciences; 2 Department of Psychology, University of Turin, Turin, Italy

## Abstract

**Introduction:**

Adverse childhood experiences (ACEs) are widely recognized as risk factors for the development of several psychiatric disorders. Among them, neglect is the least studied form of childhood maltreatment. Even less is known about its association with bipolar disorder (BD), a condition characterized by strong biological underpinnings and high familial loading. To date, no meta-analytic work has specifically addressed this relationship.

**Objectives:**

Primary aim was to evaluate the association between BD and the three main types of childhood neglect, including unspecified (Ne), physical (PN), and emotional (EN), by estimating the Odds Ratios (ORs). Secondary aim was to explore potential moderators of these associations using meta-regression.

**Methods:**

A systematic search of PubMed, Scopus, and Web of Science was conducted in September 2024, in accordance with PRISMA guidelines. Studies were eligible if they included adult populations with BD and documented exposure to childhood neglect assessed with validated instruments. ORs were pooled using random-effects models; heterogeneity was assessed with the Cochran’s Q and I² statistic, and multiple meta-regression analyses were performed to explore moderating factors.

**Results:**

Eleven studies met inclusion criteria. Childhood neglect was significantly associated with BD across different subtypes: Ne (OR = 5.89, 95% CI [1.24, 27.90]; I² = 49.2%, p = 0.139), EN (OR = 3.68, 95% CI [1.44,9 .41]; I² = 85.0%, p < 0.001), and PN (OR = 2.80, 95% CI [1.21, 6.46]; I² = 86.3%, p < 0.001) all showed strong associations. High heterogeneity was observed for EN and PN, indicating substantial between-study variability.

**Conclusions:**

The literature increasingly recognizes the role of childhood neglect as a factor that worsens the clinical trajectory of BD, contributing to earlier onset and higher psychiatric comorbidity. Our findings confirm a strong association between neglect and BD, suggesting that neglect amplifies the inherent biological vulnerability of the disorder and leads to a more severe and complex clinical course. Routine assessment of neglect may help guide preventive strategies and tailored interventions for individuals with BD.

**Disclosure of Interest:**

None Declared

